# Development of Technetium-99m-Labeled BODIPY-Based Probes Targeting Lipid Droplets Toward the Diagnosis of Hyperlipidemia-Related Diseases

**DOI:** 10.3390/molecules24122283

**Published:** 2019-06-19

**Authors:** Yoichi Shimizu, Keiichi Tanimura, Shimpei Iikuni, Hiroyuki Watanabe, Hideo Saji, Masahiro Ono

**Affiliations:** 1 Department of Patho-Functional Bioanalysis, Graduate School of Pharmaceutical Sciences, Kyoto University, 46–29, Yoshida Shimoadachi-cho, Sakyo-ku, Kyoto 606-8501, Japan; tanimura.keiichi.35z@st.kyoto-u.ac.jp (K.T.); iikuni@pharm.kyoto-u.ac.jp (S.I.); hwatanabe@pharm.kyoto-u.ac.jp (H.W.); hsaji@pharm.kyoto-u.ac.jp (H.S.); 2 Department of Diagnostic Imaging and Nuclear Medicine, Graduate School of Medicine, Kyoto University, 54 Kawahara-cho, Shogoin, Sakyo-ku, Kyoto 606-8507, Japan

**Keywords:** technetium-99m, hyperlipidemia, hydroxamamide, BODIPY

## Abstract

Hyperlipidemia causes systemic lipid disorder, which leads to hepatic steatosis and atherosclerosis. Thus, it is necessary to detect these syndromes early and precisely to improve prognosis. In the affected regions, abnormal formation and growth of lipid droplets is observed; therefore, lipid droplets may be a suitable target for the diagnosis of hyperlipidemia-related syndromes. In this study, we designed and synthesized [^99m^Tc]Tc-BOD and [^99m^Tc]Tc-MBOD composed of one technetium-99m and two BODIPY scaffolds with hydroxamamide (Ham) or *N*-methylated hydroxamamide (MHam) in radiochemical yields of 54 and 35%, respectively, with a radiochemical purity of over 95%. [^99m^Tc]Tc-BOD showed significantly higher accumulation levels in foam cells than in non-foam cells (foam cells: 213.8 ± 64.8, non-foam cell: 126.2 ± 26.9 %dose/mg protein, *p* < 0.05) 2 h after incubation. In contrast, [^99m^Tc]Tc-MBOD showed similar accumulation levels in foam cells and non-foam cells (foam cells: 92.2 ± 23.3, non-foam cell: 83.8 ± 19.8 %dose/mg protein). In normal mice, [^99m^Tc]Tc-BOD exhibited gradual blood clearance (0.5 h: 4.98 ± 0.35, 6 h: 1.94 ± 0.12 %ID/g) and relatively high accumulation in the liver 6 h after administration (15.22 ± 1.72 %ID/g). Therefore, [^99m^Tc]Tc-BOD may have potential as an imaging probe for detecting lipid droplets in disease lesions of hyperlipidemia.

## 1. Introduction

Hyperlipidemia or dyslipidemia is a chronic lipid disorder in which the lipid content in blood, such as cholesterol, increases abnormally [1]. This disease causes systemic lipid disorder, with manifestations such as fatty infiltration of the liver and lipid deposition in vessels, which leads to hepatic steatosis and atherosclerosis [2,3]. Such hyperlipidemia-related syndromes are risk factors for severe diseases, such as liver dysfunction, liver cancer, and cerebral and myocardial infarction [4,5]. Therefore, it is necessary to detect these syndromes early and precisely to improve prognosis. 

Lipid droplets are an intracellular organelle where neutral lipids of cells are stored [6,7]. In hepatic steatosis and atherosclerosis lesions, the abnormal formation and growth of lipid droplets is observed, and this is suspected to play a key role in the progression of these syndromes [8,9]. Thus, abnormally enlarged lipid droplets may be a suitable target for the diagnosis of hyperlipidemia-related syndromes. To detect lipid droplets, several hydrophobic dyes, including fluorescent probes such as Nile red and boron dipyrromethene (BODIPY), have been reported for histological or microscopic visualization [10,11]. Among these dyes, BODIPY493/503 demonstrated specific staining of lipid droplets and has been examined as an *in vitro* staining agent for the detection of lipid droplets in living cells [12]. For non-invasive *in vivo* imaging and clinical diagnosis, imaging modalities, such as positron emission tomography (PET), single photon emission computed tomography (SPECT), and magnetic resonance imaging (MRI), are widely used [13]. Although several MRI probes based on BODIPY scaffolds have been developed for the diagnosis of vulnerable plaques [14,15], no PET or SPECT imaging probe targeting lipid droplets has been reported to exist.

Therefore, we aimed to develop novel nuclear medical imaging probes based on BODIPY scaffolds for the visualization of lipid droplet regions in hyperlipidemia-related syndrome. Technetium-99m (^99m^Tc) is one of the major radioisotopes for clinical SPECT imaging because the gamma rays emitted from ^99m^Tc with a photon energy of 141-keV are readily detectable by the SPECT scanner, and ^99m^Tc is easily produced using a molybdenum-99/technetium-99m generator. To label the BODIPY scaffold with ^99m^Tc, we selected two types of chelators: hydroxamamide (Ham) and *N*-methylated hydroxamamide (MHam). Ham is a thiol-free chelating agent that produces the ^99m^Tc-complex with a high radiochemical yield under moderate reaction conditions [16,17]. In addition, Ham forms the ^99m^Tc-complex with a metal-to-ligand ratio of 1:2, which makes it possible to easily produce ^99m^Tc-labeled compounds with a bivalent targeting scaffold [18,19]. MHam is also a thiol-free chelating agent with similar properties to Ham, and the ^99m^Tc-complex with MHam exhibited higher stability in murine plasma than that with Ham [20,21]. 

In this study, we synthesized [^99m^Tc]Tc-Ham-BODIPY ([^99m^Tc]Tc-BOD) and [^99m^Tc]Tc-MHam-BODIPY ([^99m^Tc]Tc-MBOD) and evaluated their effectiveness for the detection of lipid droplets.

## 2. Results

### 2.1. Chemistry

**Scheme 1:**
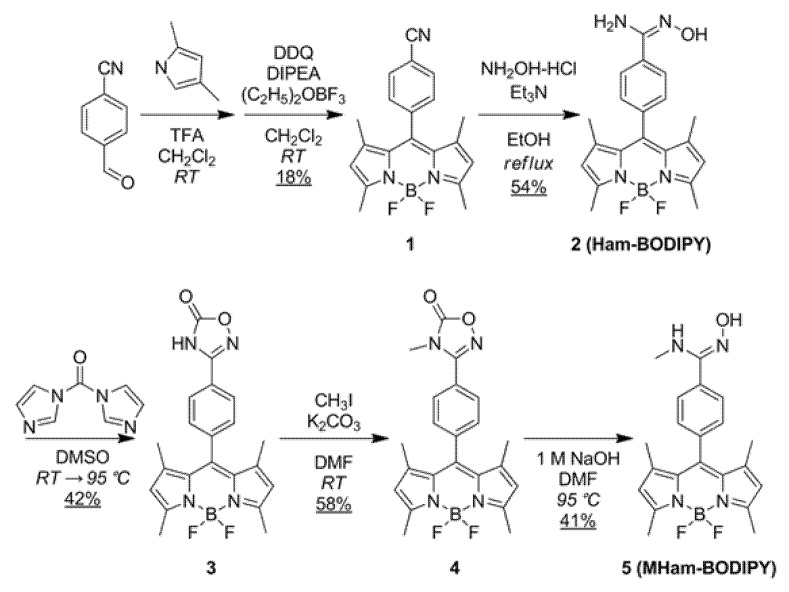
Synthetic route for Ham-BODIPY and MHam-BODIPY.

The synthesis of Ham-BODIPY (**2**) and MHam-BODIPY (**5**) is outlined in Scheme 1. The BODIPY derivative **1** was synthesized thorugh the reaction of 4-formylbenzonitrile, 2,4-dimethylpyrrole, and boron trifluoride diethyl ether complex. A Ham group was introduced into **1** through a reaction with hydroxylamine, performed according to a previously reported method This produced Ham-BODIPY (**2**) from 4-formylbenzonitrile, with a yield of 9.7% [18]. MHam-BODIPY (**5**) was prepared in three steps from **2**. Reaction of **2** with 1,1-carbonyldiimidazole provided compound **3** in DMSO. Then, **3** was methylated by iodomethane to give compound **4**. Finally, **4** was decyclized by heating in an alkaline solution to MHam-BODIPY (**5**), with the overall yield of 1.0% from 4-formylbenzonitrile.

### 2.2. Radiolabeling

**Scheme 2:**
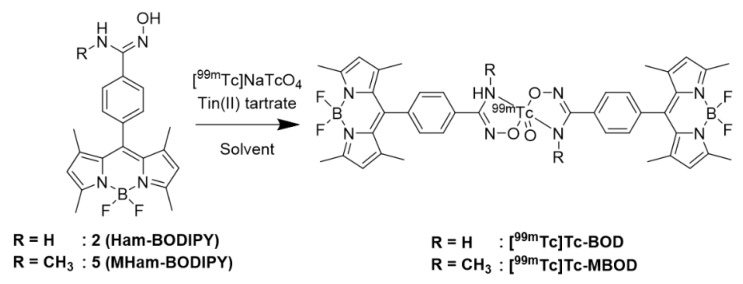
^99m^Tc labeling route and proposed chemical structures of [^99m^Tc]Tc-BOD and [^99m^Tc]Tc-MBOD.

**Figure 1:**
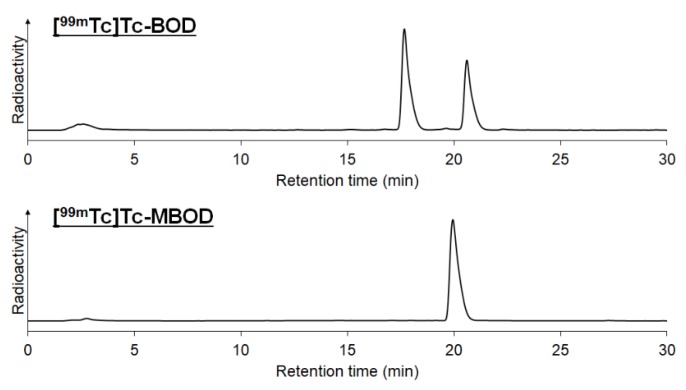
Radiochromatograms for [^99m^Tc]Tc-BOD and [^99m^Tc]Tc-MBOD after purification.

The ^99m^Tc labeling reaction was performed by the complexation reaction using Ham-BODIPY (**2**) or MHam-BODIPY (**5**), ^99m^Tc pertechnetate, and tin(II) tartrate hydrate as a reducing agent (Scheme 2). [^99m^Tc]Tc-BOD and [^99m^Tc]Tc-MBOD were obtained in a radiochemical yield of a 54 and 35%, respectively, with a radiochemical purity of over 95% (Figure 1).

### 2.3. In Vitro Cellular Uptake Study

**Figure 2:**
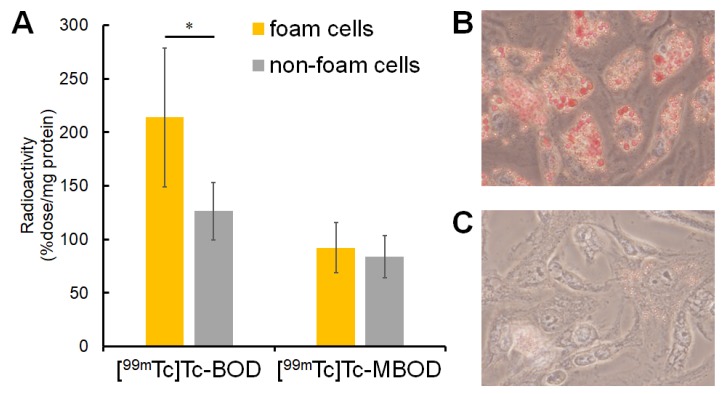
(**A**) *In vitro* cellular uptake of [^99m^Tc]Tc-BOD and [^99m^Tc]Tc-MBOD. Values are expressed as the mean ± standard error of six independent experiments. * *p* < 0.05 (Student’s *t*-test). (**B**,**C**) Oil red O staining images of (**B**) foam cells and (**C**) non-foam cells. Deep red staining of **B** indicates the rich formation of lipid droplets.

The cellular uptake of [^99m^Tc]Tc-BOD and [^99m^Tc]Tc-MBOD is expressed as the %dose/mg protein (Figure 2A), and foam cell formation was confirmed using Oil red O staining (Figure 2B,C). The cellular uptake of [^99m^Tc]Tc-BOD into foam cells was significantly higher than that into non-foam cells (foam cell: 213.8 ± 64.8, non-foam cell: 126.2 ± 26.9 %dose/mg protein, *p* < 0.05). On the other hand, the difference between the uptake of [^99m^Tc]Tc-MBOD into foam cells and non-foam cells was not significant (foam cell: 92.2 ± 23.3, non-foam cell: 83.8 ± 19.8 %dose/mg protein) (Figure 2A).

### 2.4. Stability in Murine Plasma

**Figure 3:**
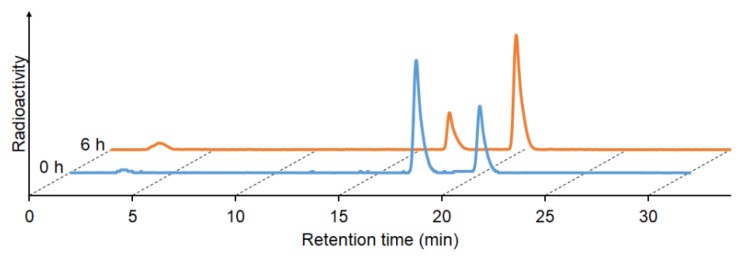
*In vitro* stability of [^99m^Tc]Tc-BOD in murine plasma. [^99m^Tc]Tc-BOD had a radiochemical purity of over 80% in murine plasma 6 h after incubation.

The *in vitro* stability of [^99m^Tc]Tc-BOD was evaluated by incubation in murine plasma at 37 °C for 6 h. [^99m^Tc]Tc-BOD had a radiochemical purity of over 80% in murine plasma 6 h after incubation (Figure 3).

### 2.5. In Vivo Biodistribution

The uptake of [^99m^Tc]Tc-BOD in each organ was expressed as the %ID/g, except for in the stomach, which was expressed as the %ID (Table 1). [^99m^Tc]Tc-BOD exhibited gradual blood clearance (0.5 h: 4.98 ± 0.35, 6 h: 1.94 ± 0.12 %ID/g) and demonstrated relatively higher uptake in the liver (6 h: 15.22 ± 1.72 %ID/g) and stomach (6 h: 25.25 ± 5.30 %ID).

## 3. Discussion

In this study, we developed novel ^99m^Tc labeled probes, [^99m^Tc]Tc-BOD and [^99m^Tc]Tc-MBOD, for the purpose of imaging of lipid droplets. Ham-BODIPY (**2**) and MHam-BODIPY (**5**), the precursors of [^99m^Tc]Tc-BOD and [^99m^Tc]Tc-MBOD, respectively, were synthesized with total yields of 9.7 and 1.0%, respectively (Scheme 1). [^99m^Tc]Tc-BOD and [^99m^Tc]Tc-MBOD were obtained under moderate conditions (at room temperature for 10 min) with radiochemical yields of 54 and 35%, respectively. In general, the chelation reactions of technetium-99m and hydroxamamide/N-methylated hydroxamamide have been evaluated only by confirming the disappearance of the peak derived from [^99m^Tc]TcO_4_^−^ in HPLC analysis. This is because it remains challenging to synthesize the analogues of the rhenium hydroxamamide/N-methylated hydroxamamide complexes [18,19]. In this study, when the reaction mixture of Ham-BODIPY (**2**) was analyzed using RP-HPLC, two ^99m^Tc-labeled compounds were found at retention times of 17.7 and 20.6 min ([^99m^Tc]Tc-BOD), which were different from the retention time of [^99m^Tc]NaTcO_4_ (Supplementary Figure S1E). This result was; consistent with previous reports on ^99m^Tc-Ham complexes, suggesting that two isomers of [^99m^Tc]Tc-BOD were produced by the ^99m^Tc complexation reaction using Ham (Figure 1) [18]. There is no report stating that BODIPY itself is a ligand or substrate for specific receptors or enzymes, and it is suspected to be delivered to lipid droplets via its lipophilicity. Thus, we considered the two isomers of [^99m^Tc]Tc-BOD not to have different properties for targeting lipid droplets, and we used a mixture of both isomers for the *in vitro* and *in vivo* evaluations of [^99m^Tc]Tc-BOD. On the other hand, analysis of the ^99m^Tc complexation reaction with MHam-BODIPY (**5**) by RP-HPLC revealed a single radioactive peak at a retention time of 20.0 min ([^99m^Tc]Tc-MBOD). This suggested that N-methylation of Ham is a useful strategy to obtain a single ^99m^Tc-MHam complex by preventing isomerism, as described in a previous report [20]. 

We next performed an *in vitro* study to elucidate whether [^99m^Tc]Tc-BOD and [^99m^Tc]Tc-MBOD are able to detect lipid droplets. As lipid-rich model cells, we selected foam macrophages, which are used for studies on atherosclerosis [22,23]. Previously reported optical imaging probes have hydrophobic characteristics to detect lipid droplets [10]. [^99m^Tc]Tc-BOD accumulation in foam cells was significantly higher than that in non-foam cells, suggesting that [^99m^Tc]Tc-BOD may be taken up specifically by cells with lipid droplets. However, [^99m^Tc]Tc-MBOD accumulated similarly in both foam cells and non-foam cells. The reason as to why [^99m^Tc]Tc-MBOD did not accumulate in foam cells specifically is still unclear, but the hydrophobicity of the BODIPY scaffold may not be as necessary for specific accumulation in foam cells. 

We next evaluated the distribution of [^99m^Tc]Tc-BOD in normal (ddY) mice. In murine plasma, although the rate of the two [^99m^Tc]Tc-BOD isoforms was changed, [^99m^Tc]Tc-BOD exhibited high stability for 6 h, which is suitable for *in vivo* imaging (Figure 3). In normal mice, [^99m^Tc]Tc-BOD levels gradually decreased in the blood, which is suitable for SPECT imaging (Table 1). However, [^99m^Tc]Tc-BOD also had relatively high uptake and retention in the stomach and liver. To visualize hepatic steatosis, [^99m^Tc]Tc-BOD (with its high uptake and retention in the liver) is not suitable. One strategy to solve this problem is to connect two Ham groups with a propylene linker, which was reported to reduce liver retention in a previous report [24]. Regarding the diagnosis of atherosclerosis, some previously reported probes also had high accumulation in the liver [25]. Therefore, [^99m^Tc]Tc-BOD holds potential for imaging lipid droplets in atherosclerotic lesions.

Several kinds of probes labeled with fluorine-18 and iodine-123/125 have been reported as radiolabeled probes with a BODIPY scaffold [26,27,28]. However, fluorine-18 is a cyclotron-produced isotope; this limits the production of fluorine-18-labeled probes because installation of a cyclotron system is expensive and requires a large facility. On the other hand, ^99m^Tc is easily produced using a molybdenum-99/technetium-99m generator without a large facility, and probes labeled with ^99m^Tc can be produced via a one-step reaction with ^99m^Tc. Some BODIPY-based compounds labeled with ^99m^Tc have been reported [11]. Compared with those compounds, the radiolabeling condition of [^99m^Tc]Tc-BOD is mild (only requiring incubation for 10 min at room temperature), which makes it more convenient for the acquisition of ^99m^Tc-labeled BODIPY. Thus, [^99m^Tc]Tc-BOD may be useful as a radiolabeled probe with a BODIPY scaffold.

A multimodal imaging technique combining nuclear medical imaging and fluorescence imaging has been developed. It is expected to integrate each advantage, such as a high spatial resolution in fluorescence imaging and a high signal permeability in nuclear medical imaging [29]. The BODIPY scaffold we used as a ligand targeting lipid droplets has suitable properties for fluorescence imaging (Supplementary Table S1 and Figure S2). Therefore, we tried to perform a microscopic fluorescence imaging study. In this study, we used Ham-BODIPY instead of [^99m^Tc]Tc-BOD because it is quite difficult to acquire enough ^99m^Tc-radiolabeled compound (i.e., approximately a femtomole level) for fluorescence imaging (Supplementary Figure S3). As a result, a high florescent signal was observed in the foam cells treated with Ham-BODIPY (Supplementary Figure S3B), whereas the fluorescent signal was observed not only in the lipid droplets but also in the other cell components. This was also observed in the non-foam cells treated with Ham-BODIPY. Although the chemical properties of [^99m^Tc]Tc-BOD and Ham-BODIPY presumed from the HPLC analysis were different (Figure 1 and Supplementary Figure S1), this result may correlate with the non-specific accumulation of [^99m^Tc]Tc-BOD in the non-foam cells (Figure 2A). Therefore, we are now trying to optimize ^99m^Tc-labeled BODIPY probes for achieving more specific imaging of lipid droplets.

## 4. Materials and Methods 

### 4.1. General

All reagents were obtained commercially and used without further purification unless otherwise indicated. [^99m^Tc]NaTcO_4_ was purchased from Nihon Medi-Physics Co., Ltd. (Tokyo, Japan). W-Prep 2XY (Yamazen, Osaka, Japan) was used for silica gel column chromatography on a Hi Flash silica gel column (40 μm, 60 Å, Yamazen). ^1^H-NMR and ^13^C-NMR spectra were recorded on a JNM-ECS400 (JEOL, Tokyo, Japan) with tetramethylsilane as an internal standard. Coupling constants are reported in hertz. Multiplicity was defined as singlet (s), doublet (d), or multiplet (m). Mass spectra were obtained using a SHIMADZU LCMS-2020 (SHIMADZU, Kyoto, Japan). RP-HPLC was performed with a Shimadzu system (SHIMADZU, an LC-20AT pump with an SPD-20A UV detector, λ = 254 nm) with a Cosmosil C_18_ column (Nacalai Tesque, Kyoto, Japan, 5C_18_-AR-II, diameter: 4.6 mm, length: 150 mm, particle size: 5 μm, pore size: 120 Å). 

### 4.2. Chemistry

#### 4.2.1. Synthesis of **1**

Trifluoroacetic acid (TFA) (30 μL) and 2,4-dimethylpyrrole (1.00 g, 10.5 mmol) were added to a solution of 4-formylbenzonitrile (655 mg, 5 mmol) in dichloromethane (40 mL). The reaction mixture was stirred at room temperature for 6 h. To the reaction mixture, 2,3-dichloro-5,6-dicyano-p-benzoquinone (DDQ) (1.14 g, 5 mmol), *N,N*-diisopropylethylamine (DIEPA) (10 mL), and boron trifluoride diethyl ether complex were added. The reaction mixture was then stirred at room temperature overnight. After the addition of water (80 mL), the mixture was extracted with dichloromethane (40 mL × 2). The organic layers were combined, dried over MgSO_4_, and filtered. Evaporation of the filtrate resulted in the formation of a residue, which was purified using silica gel chromatography (chloroform/hexane = 3/2) to give 313 mg of **1** (18% yield). ^1^H-NMR (400 MHz, CDCl_3_) δ 1.36 (s, 6H), 2.56 (s, 6H), 6.01 (s, 2H), 7.47 (d, *J* = 8.0 Hz, 2H), 7.82 (d, *J* = 8.0 Hz, 2H). ^13^C-NMR (100 MHz, CDCl_3_) δ14.6 (4C), 113.2 (2C), 118.1 (1C), 121.8 (2C), 129.3 (2C), 130.7 (1C), 132.9 (2C), 138.6 (1C), 140.0 (2C), 142.5 (2C), 156.5 (2C). MS (ESI, pos) *m*/*z* 350.2 [M + H]^+^.

#### 4.2.2. Synthesis of **2** (Ham-BODIPY)

Hydroxylamine hydrochloride (188 mg, 2.7 mmol) and triethylamine (374 μL, 2.7 mmol) were added to a solution of **1** (313 mg, 0.9 mmol) in ethanol (20 mL). The reaction mixture was heated to reflux overnight. After the addition of water (60 mL), the mixture was extracted with chloroform (60 mL × 2). The organic layers were combined, dried over MgSO_4_, and filtered. Evaporation of the filtrate gave a residue, which was purified by silica gel chromatography (ethyl acetate/hexane = 9/1) to give 187 mg of 2 (Ham-BODIPY) (54% yield). ^1^H-NMR (400 MHz, dimethyl sulfoxide (DMSO)-*d_6_*) δ1.38 (s, 6H), 2.46 (s, 6H), 5.95 (s, 2H), 6.19 (s, 2H), 7.38 (d, *J* = 8.4 Hz, 2H), 7.88 (d, *J* = 8.4 Hz, 2H), 9.81 (s, 1H). ^13^C-NMR (100 MHz, DMSO-*d_6_*) δ14.1 (2C), 14.2 (2C), 121.4 (2C), 126.0 (2C), 127.7 (2C), 130.6 (2C), 133.9 (1C), 134.4 (2C), 141.6 (1C), 142.7 (1C), 150.0 (2C), 154.9 (2C). High-resolution (HR)-MS (FAB, pos) *m*/*z* calcd. 383.1855, found 383.1858 [M + H]^+^.

#### 4.2.3. Synthesis of **3**

To a solution of **2** (104 mg, 0.30 mmol) in DMSO (10 mL), 1,1-carbonyldiimidazole (58 mg, 0.36 mmol) was added. The reaction mixture was stirred and heated gradually from room temperature to 95 °C for 6 h. The reaction mixture was lyophilized and purified by silica gel chromatography (chloroform/methanol = 9/1) to give 46 mg of 3 (42% yield). ^1^H-NMR (400 MHz, CDCl_3_) δ1.38 (s, 6H), 2.56 (s, 6H), 5.99 (s, 1H), 7.10 (s, 1H), 7.42 (d, *J* = 8.4 Hz, 2H), 8.03 (d, *J* = 8.4 Hz, 2H). ^13^C-NMR (100 MHz, CDCl_3_) δ14.6 (4C), 121.5 (2C), 125.2 (1C), 126.9 (2C), 128.4 (1C), 128.5 (1C), 129.0 (2C), 131.0 (1C), 138.5 (2C), 139.9 (1C), 142.8 (2C), 156.0 (2C). MS (ESI, pos) *m*/*z* 409.2 [M + H]^+^.

#### 4.2.4. Synthesis of **4**

Iodomethane (86 μL, 1.38 mmol) and potassium carbonate (95 mg, 0.69 mmol) were added to a solution of **3** (94 mg, 0.23 mmol) in *N,N*-dimethylformamide (DMF) (20 mL). The reaction mixture was stirred at room temperature for 3 h. After the addition of water (50 mL), the mixture was extracted with chloroform (30 mL × 2). The organic layers were combined, dried over MgSO_4_, and filtered. Evaporation of the filtrate gave a residue, which was purified by silica gel chromatography (chloroform/methanol = 9/1) to give 57 mg of **4** (58% yield). ^1^H-NMR (400 MHz, CDCl_3_) δ1.38 (s, 6H), 2.56 (s, 6H), 3.39 (s, 3H), 6.02 (s, 2H), 7.54 (d, *J* = 8.4 Hz, 2H), 7.80 (d, *J* = 8.4 Hz, 2H). ^13^C-NMR (100 MHz, CDCl_3_) δ14.5 (2C), 14.6 (2C), 29.9 (1C), 121.7 (1C), 124.0 (2C), 128.2 (1C), 128.8 (2C), 129.4 (2C), 139.0 (1C), 139.2 (1C), 142.5 (1C), 156.3 (2C), 158.0 (2C), 159.5 (2C). MS (ESI, pos) *m*/*z* 423.2 [M + H]^+^.

#### 4.2.5. Synthesis of **5** (MHam-BODIPY)

A solution of **4** (43 mg, 0.10 mmol) in 1 M NaOH (aq)/DMF (1/1, 20 mL) was stirred at 95 °C for 14 h. The reaction mixture was neutralized with 1 M HCl (aq) while being cooled in an ice bath. After extraction with chloroform, the organic layers were combined, dried over MgSO_4_, and filtered. The filtrate was concentrated, and the residue was purified by silica gel chromatography (chloroform/methanol = 9/1) to give 18 mg of **5** (41% yield). ^1^H-NMR (400 MHz, DMSO-*d_6_*) δ1.38 (s, 6H), 2.46 (s, 6H), 2.60 (d, *J* = 5.2 Hz, 3H), 5.87 (m, 1H), 6.20 (s, 2H), 7.42 (d, *J* = 8.0 Hz, 2H), 7.80 (d, *J* = 8.0 Hz, 2H), 9.68 (s, 1H). ^13^C-NMR (100 MHz, DMSO-*d_6_*) δ14.0 (2C), 14.2 (2C), 30.3 (1C), 121.5 (1C), 127.8 (2C), 129.1 (2C), 130.6 (1C), 133.4 (2C), 134.4 (2C), 141.4 (1C), 142.7 (1C), 154.8 (2C), 155.0 (2C). HR-MS (FAB, pos) *m*/*z* calcd. 397.2011, found 397.2016 [M + H]^+^.

### 4.3. Radiolabeling

To a solution of **2** (Ham-BODIPY) or **5** (MHam-BODIPY) (1 mg) in an acetic acid/ethanol mixture (1/4, 100 μL), 100 μL of [^99m^Tc]NaTcO_4_ solution (37 MBq) and 30 μL of tin(II) tartrate hydrate solution (2 mg of tin(II) tartrate hydrate (7.5 μmol) dissolved in water (2.5 mL)) were added. The reaction mixture was incubated at room temperature for 10 min and purified by RP-HPLC on a Cosmosil 5C_18_-AR-II column (Nacalai Tesque, diameter: 4.6 mm, length: 150 mm, particle size: 5 μm, pore size: 120 Å) using the mobile phase (10 mM phosphate buffer (pH of 7.4)/acetonitrile = 60/40 (0 min) to 30/70 (30 min)) at a flow rate of 1.0 mL/min. To acquire [^99m^Tc]Tc-BOD, the two main peaks (retention time: around 17 to 18 min and around 20 to 21 min) were collected, combined, and then concentrated to remove acetonitrile. The mixture of those two fractions was used as [^99m^Tc]Tc-BOD in the subsequent examination. As for [^99m^Tc]Tc-MBOD, the main peak (retention time: around 20 to 21 min) was collected, then concentrated. The radiochemical yields of [^99m^Tc]Tc-BOD and [^99m^Tc]Tc-MBOD were calculated by dividing the radioactivity of the collected fraction (the mixture of the two peaks in the radiolabeling study of [^99m^Tc]Tc-BOD) by the radioactivity of the cludes measured before HPLC purification. The radiochemical purities were analyzed using RP-HPLC with the same method as the purification procedures.

### 4.4. Preparation of Foam Cells

Animal experiments were conducted in accordance with our institutional guidelines and approved by the Kyoto University Animal Care Committee. ddY mice were purchased from Japan SLC, Inc. (Shizuoka, Japan) and were housed with food and water supplied ad libitum under a 12-h light/12-h dark cycle. To serve as lipid droplet-rich model cells, we prepared mouse foam cells following a previously reported method [22]. In brief, 10% thioglycolate medium (10 mL) was injected intraperitoneally to ddY mice (females, 5 weeks old). After being housed for 4 days, the mice were sacrificed and the peritoneal cavity was washed using an injection of phosphate-buffered saline (PBS) (pH of 7.4) (Nacalai Tesque, Kyoto, Japan) to isolate macrophages. The macrophages were plated on 6-well plates (8 × 10^5^ cells/well) and incubated in Dulbecco’s modified Eagle’s medium (DMEM) (4500 mg/L of Glucose; Nacalai Tesque) supplemented with 10% fetal bovine serum and 100 U/mL of penicillin and streptomycin at 37 °C in an atmosphere containing 5% CO_2_ for 24 h. After incubation, the medium was removed and acetyl low density lipoprotein (LDL) (50 μg/mL) was added to the new medium (2 mL). Macrophages were incubated to form foam cells for 48 h. The formation of foam cells was confirmed by Oil red O staining. After removing the medium and washing twice with 1 mL of PBS (pH of 7.4), 2 mL of staining solution (Oil red O 1.8 mg/mL in 2-propanol/water = 3/2) was added to each well, and incubated at room temperature for 20 min. The staining solution was removed, and cells were washed twice with 1 mL of PBS (pH of 7.4). Bright field images were obtained using FSX-100 (Olympus, Tokyo, Japan).

### 4.5. In Vitro Cellular Uptake Study

[^99m^Tc]Tc-BOD (the mixtures of two isomers) or [^99m^Tc]Tc-MBOD (37 kBq, 2 mL in DMEM supplemented with 0.1% DMSO) was added to foam cells or non-foam cells cultured in 6-well plates (*n* = 6), and the plates were incubated at 37 °C in an atmosphere containing 5% CO_2_ for 2 h. After incubation, each well was washed with 1 mL of PBS (pH of 7.4) and the cells were lysed with 1 M NaOH (0.2 mL × 2). The radioactivity of the cells was measured using a γ counter (Wallac 2470 Wizard; PerkinElmer, Waltham, MA, USA). The protein concentration was measured using a bicinchoninic acid protein assay kit (Thermo Fisher Scientific, Waltham, MA, USA).

### 4.6. Stability in Murine Plasma

[^99m^Tc]Tc-BOD (the mixtures of two isomers; 740 kBq) was added to freshly prepared murine plasma (200 μL) collected from ddY mice (male, 5 weeks old). After incubating the solution at 37 °C for 6 h, acetonitrile (200 μL) was added. Subsequently, the solution was centrifuged (4,000 *g*, 5 min) and the supernatant was analyzed using RP-HPLC. The analytical method of RP-HPLC was the same as that described in the radiolabeling section.

### 4.7. In Vivo Biodistribution

Male ddY mice (5 weeks old) were purchased from Japan SLC. Inc. (Shizuoka, Japan). [^99m^Tc]Tc-BOD (the mixtures of two isomers) (480 kBq, 200 μL in saline supplemented with 0.1% DMSO) was injected into ddY mice via the tail vein. After injection, the mice were sacrificed at 0.5, 1, 3, or 6 h post-injection (*n* = 5, per time-point). The blood was collected, organs of interest (blood, spleen, pancreas, stomach, intestine, kidney, liver, heart, and lung) were dissected and weighed, and the radioactivity in each organ or tissue was measured using a γ counter (PerkinElmer). The results are expressed as a percentage of the injected dose per gram (%ID/g) for the selected organs, except the stomach whose result is expressed as a percentage of the injected dose (%ID).

### 4.8. Statistics

The student’s *t*-test was used to assess the significance of differences. Differences at the 95% confidence level (*p* < 0.05) were considered significant.

## 5. Conclusions

In this study, we developed [^99m^Tc]Tc-BOD and [^99m^Tc]Tc-MBOD as novel BODIPY-based probes radiolabeled with ^99m^Tc. [^99m^Tc]Tc-BOD exhibited higher accumulation in foam cells and suitable properties for *in vivo* imaging. Thus, the [^99m^Tc]Tc-BOD may have potential as an imaging probe to detect lipid droplets.

## Figures and Tables

**Table 1:** Biodistribution of [^99m^Tc]Tc-BOD in normal (ddY) mice.

Organ	Time after Injection			
	0.5 h	1 h	3 h	6 h
Blood	4.98 ± 0.35	3.33 ± 0.29	2.70 ± 0.33	1.94 ± 0.12
Spleen	6.46 ± 1.09	5.47 ± 1.34	5.44 ± 0.79	5.58 ± 0.65
Pancreas	1.41 ± 0.15	1.08 ± 0.17	0.96 ± 0.16	0.79 ± 0.10
Stomach*	23.87 ± 2.56	28.77 ± 3.52	29.80 ± 3.48	25.25 ± 5.30
Intestine	2.03 ± 0.31	2.94 ± 0.41	5.28 ± 0.37	7.90 ± 1.72
Kidney	2.43 ± 0.30	2.16 ± 0.35	2.07 ± 0.26	2.07 ± 0.39
Liver	17.43 ± 1.87	14.99 ± 2.63	15.93 ± 1.32	15.22 ± 1.72
Heart	1.96 ± 0.15	1.22 ± 0.14	1.19 ± 0.13	0.89 ± 0.08
Lung	8.85 ± 2.42	4.80 ± 0.82	3.22 ± 0.70	2.33 ± 0.44

Values are expressed as the % injected dose per gram (%ID/g) of organ tissue. Each value is the mean ± standard deviation of five mice at each interval. *Values are expressed as the % injected dose (%ID).

## Supplementary Materials

The Supplementary Materials are available online.
