# Supplementary material for: Development of Technetium-99m-Labeled BODIPY-Based Probes Targeting Lipid Droplets Toward the Diagnosis of Hyperlipidemia-Related Diseases
Source: Molecules. 2019 Jun 19;24(12):2283. doi: 10.3390/molecules24122283 (PMC6631856; doi:10.3390/molecules24122283)
Supplement: Supplementary file 1 [file molecules-24-02283-s001.pdf]

Supplementary Materials

Development of Technetium-99m-Labeled BODIPY-Based Probes Targeting Lipid Droplets Toward the Diagnosis of Hyperlipidemia-Related Diseases

Yoichi Shimizu [1,2,*,†], Keiichi Tanimura [1,†], Shimpei Iikuni [1], Hiroyuki Watanabe [1], Hideo Saji [1] and Masahiro Ono [1,*]

1 Department of Patho-Functional Bioanalysis, Graduate School of Pharmaceutical Sciences, Kyoto University, 46–29, Yoshida Shimoadachi-cho, Sakyo-ku, Kyoto 606-8501, Japan; tanimura.keiichi.35z@st.kyoto-u.ac.jp (K.T.); iikuni@pharm.kyoto-u.ac.jp (S.I.); hwatanabe@pharm.kyoto-u.ac.jp (H.W.); hsaji@pharm.kyoto-u.ac.jp (H.S.)

2 Department of Diagnostic Imaging and Nuclear Medicine, Graduate School of Medicine, Kyoto University, 54 Kawahara-cho, Shogoin, Sakyo-ku, Kyoto 606-8507, Japan

* Correspondence: yoichis@kuhp.kyoto-u.ac.jp (Y.S.); ono@pharm.kyoto-u.ac.jp (M.O.); Tel.: +81-75-751-3760 (Y.S.); +81-75-753-4556 (M.O.)

† These authors contributed equally to this manuscript.

Supplementary Materials and Methods

1. Optical Characteristics of Ham-BODIPY and MHam-BODIPY

Absorption spectra of Ham-BODIPY (10 μM DMSO) and MHam-BODIPY (10 μM DMSO) were measured by UV-1800 UV-VIS spectrophotometer (SHIMADZU, Kyoto, Japan). Fluorescence emission/excitation spectra and quantum yields of Ham-BODIPY (10 μM DMSO) and MHam-BODIPY (10 μM DMSO) were measured by RF-6000 fluorescence spectrometer (SHIMADZU). The quantum yields were determined by using quinine sulfate as the standard sample.

2. Fluorescence Microscopy

Mouse macrophages were acquired from ddY mice as written in the material and method section "4.4. Preparation of foam cells". The isolated macrophages (1 x 10^6 cells) were added to a glass-bottom dish, and incubated in DMEM (4,500 mg/L of Glucose; Nacalai Tesque, Kyoto, Japan) supplemented with 10% fetal bovine serum and 100 U/mL of penicillin and streptomycin at 37°C in an atmosphere containing 5% CO_2 for 24 h. After incubation, the medium was removed and acetyl low density lipoprotein (50 μg/mL) was added to new medium (2 mL). After 48-hour incubation to form foam cells, Ham-BODIPY (10 μM DMEM supplemented with 0.1% DMSO, 2 mL) was added to the cells and incubated for 2 h at 37 °C in a humidified atmosphere containing 5% CO_2. The cells were then washed twice with PBS, and were examined by fluorescence microscopy (FSX100, Olympus Corp., Tokyo, Japan) equipped with a U-MWIG3 filter set. Fluorescence images were analyzed with cellSens software (Olympus Corp.).

Supplementary Table

Supplementary Table 1. Optical properties of Ham-BODIPY and MHam-BODIPY in DMSO.

Entry	Absorption Maximum (nm)	Excitation Maximum (nm)	Emission Maximum (nm)	Quantum Yield	Extinction Coefficient (M^{-1} cm^{-1})
Ham-BODIPY	502	505	515	0.189	65,000
MHam-BODIPY	502	505	513	0.179	58,000

Supplemental Figures

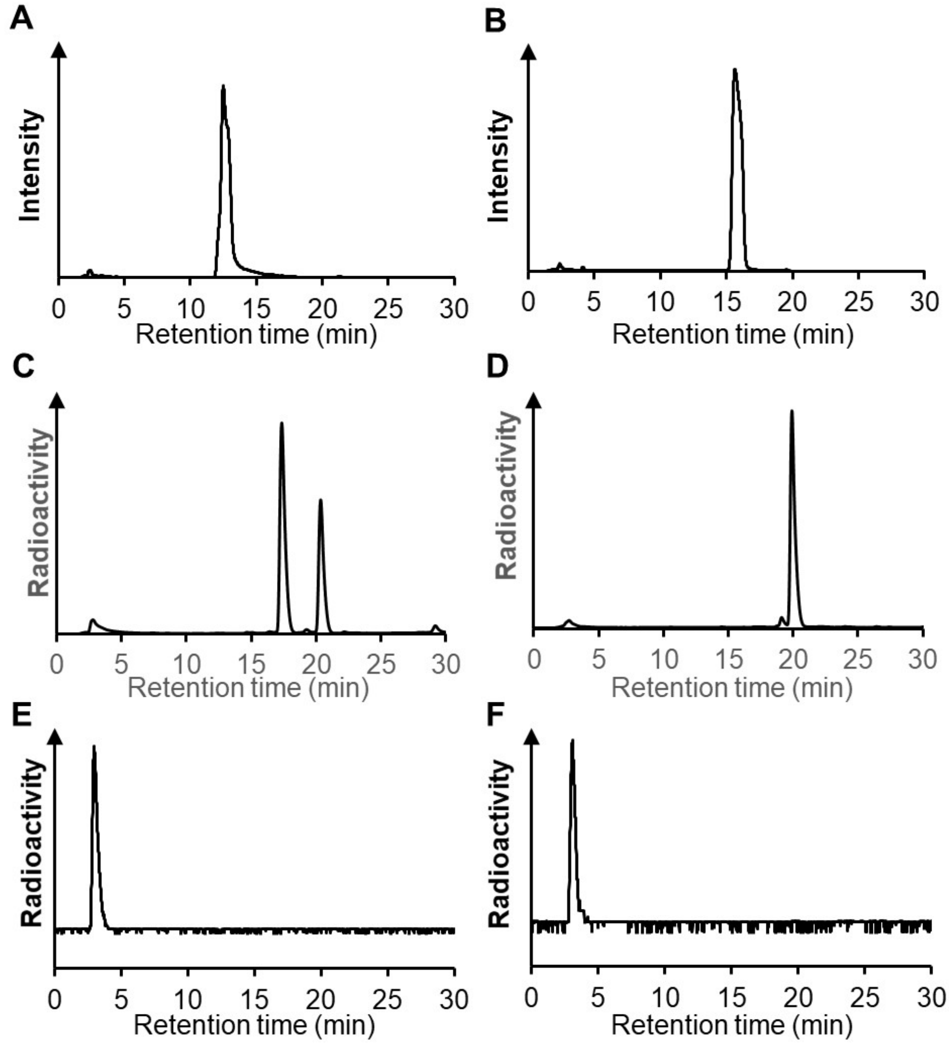

Supplementary Figure 1. A,B: Chromatograms for (A) Ham-BODIPY and (B) MHam-BODIPY. C, D: Radiochromatograms for (C) [^{99m}Tc]Tc-BOD and (D) [^{99m}Tc]Tc-MBOD before purification. E, F: Radiochromatograms for (E) [^{99m}Tc]$NaTcO_4$, and (F) the reactant of [^{99m}Tc]$NaTcO_4$ solution and tin(II) tartrate hydrate solution without precursors.

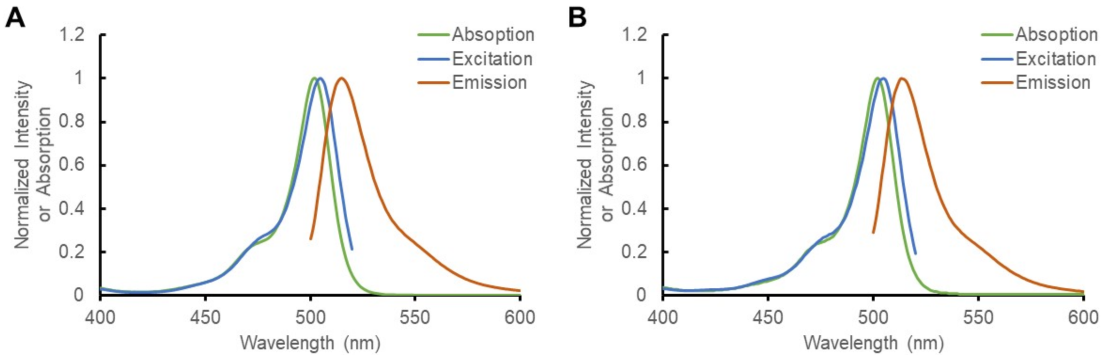

Supplementary Figure 2. Emission (red liine), excitation (blue line) and absorption (green line) spectra of (A) Ham-BODIPY and (B) MHam-BODIPY in DMSO.

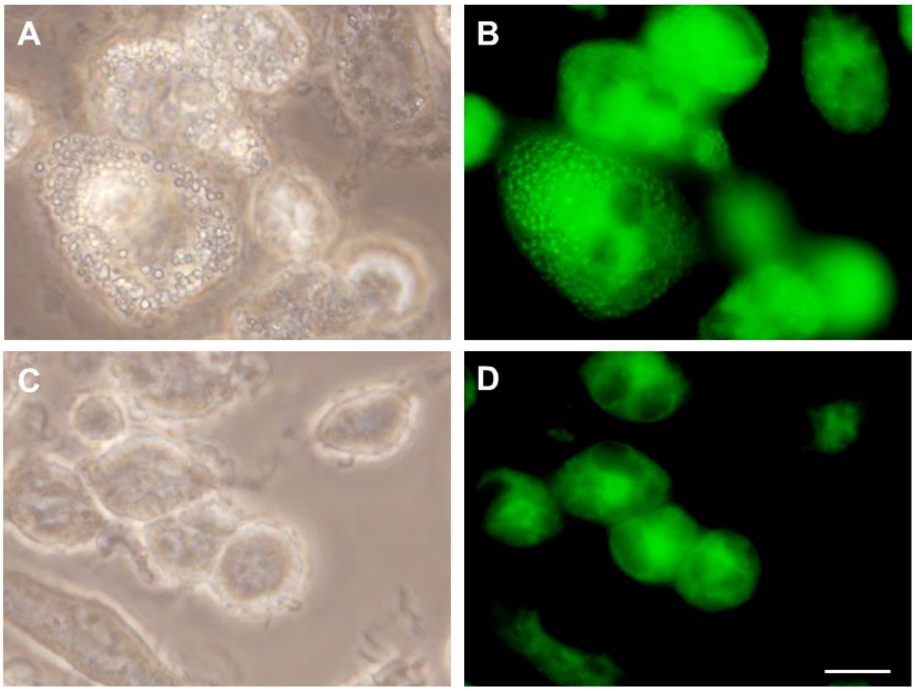

Supplementary Figure 3. Microscopic observation of (A, B) foam cells or (C, D) non-foam cells treated with Ham-BODIPY. Fluorescence images (B, D) are shown with bright-field images (A, C). Scale bar indicates 10 μm.
